# Homesickness in social context: An ecological momentary assessment study among 1st‐year university students

**DOI:** 10.1002/ijop.12586

**Published:** 2019-05-26

**Authors:** Maaike H. Nauta, Marije aan het Rot, Henk Schut, Margaret Stroebe

**Affiliations:** ^1^ Department of Psychology, Section Clinical Psychology and Experimental Psychopathology University of Groningen Groningen The Netherlands; ^2^ Department of Clinical Psychology University of Utrecht Utrecht The Netherlands

**Keywords:** Homesickness, Acculturation, Ecological momentary assessment, Social interactions, Positive and negative effect

## Abstract

Homesickness is common among university students and associated with mental health problems. Most previous studies assessed homesickness as a summary of the past weeks. However, there may be significant fluctuations across situations. At the current residence, homesickness may especially be triggered during (phone) interactions with attachment figures. Dutch and international 1st‐year students (*n* = 92) completed the Utrecht Homesickness Scale and subsequently used a smartphone application to record social interactions for 14 days (ecological momentary assessment [EMA]). For each interaction they reported the social context (e.g. location, contact type) and their affective state, including homesickness. Homesickness in the past weeks and momentary homesickness were both higher in international students than in Dutch students. Feeling homesick was highest at participants' current residency, when interacting with parents, or when using video‐chat. When participants felt more homesick, they reported less pleasant and more unpleasant affect. In conclusion, EMA provided insight in cross‐situational variations in homesickness.

Homesickness has been defined as “a negative emotional state primarily due to separation from home and attachment persons, characterized by longing for and preoccupation with home, and often with difficulties adjusting to a new place” (Stroebe, Schut, & Nauta, 2016). A growing body of literature confirms that homesickness is associated with various socioemotional difficulties, as well as with mental and physical health problems (Stroebe, Schut, & Nauta, 2015).

Homesickness among university students has been the focus of most studies, reflecting a concern of young people leaving their parental home for the first extended period in their lives (Thurber & Walton, 2012). A majority of 1st‐year students experience some degree of homesickness (Stroebe et al., 2015). While most students adjust over time, for a minority difficulties remain, sometimes of clinical significance (e.g. English, Davis, Wei, & Gross, 2017). Given the increasingly global orientation of university education, homesickness among students may become even more prominent.

Attachment theory has contributed significantly to understanding separation experiences such as leaving home, providing a framework to understand phenomena such as homesickness (e.g. Bowlby, 1969; Stroebe et al., 2015). According to this perspective, interactions with attachment figures activate the so‐called attachment behavioural system. Therefore homesickness may be felt more acutely when students interact with home‐based attachment figures, compared with people in the new environment.

Homesickness has typically been assessed by asking participants to complete a questionnaire summarising their homesickness during the past weeks. Such retrospective assessment is prone to bias (Thurber, 1995). Questionnaire scores represent past homesickness, which presumably reflects one's proneness to homesickness or overall disposition to homesickness more than one's momentary homesickness. In other words, cross‐sectional questionnaire studies draw a general picture. They do not provide a dynamic set of snapshots of a phenomenon that fluctuates over time.

While some studies have assessed homesickness once a day (Thurber, 1995), fluctuations in homesickness may even occur within smaller time periods, for example, depending on the situation. As attachment figures are central to homesickness, it can (partly) be considered an interpersonal phenomenon. Homesickness may mostly be experienced when the attachment system is activated (Bowlby, 1969), for instance, when parents are physically absent but in contact through phone conversations. Thus, within‐person levels of homesickness are expected to vary between interactions with different persons.

While homesickness has been associated with concurrent and future mental health problems (e.g. Fisher & Hood, 1987), whether this translates to associations between momentary homesickness and affective states is unclear. If experiencing homesickness is positively related to experiencing unpleasant affect and negatively related to pleasant affect, then this may put individuals who frequently miss home at risk for developing mental health problems. In line with this idea, English et al. (2017) described the negative impact of homesickness, measured weekly during the first university semester, on psychosocial adjustment at the end of the semester. Social interactions were particularly affected.

The aim of the present study was to increase insight into the nature and correlates of homesickness experienced during every‐day social interactions. We know of no prior studies examining how momentary homesickness may vary across interactions with different people. Ecological momentary assessment (EMA) of social interactions enables this, as it involves intensive repeated measures in daily life (Moskowitz & Sadikaj, 2012). Using EMA, we examined whether proneness to homesickness predicted momentary homesickness, and whether variations in momentary homesickness would be associated with variation in social context and affective state. We hypothesized that (1) international students in the Netherlands would experience more homesickness than Dutch students, (2) homesickness would be higher while participants were (i) physically away from their parental home, and (ii) interacting with people they knew before moving out. Finally, (3) feeling homesick would be associated with more unpleasant and less pleasant affect.

## METHODS

### Ethics statement

The study was reviewed by the departmental Ethics Committee and conducted in accordance with the Declaration of Helsinki. All participants provided written informed consent.

### Participants

First‐year students of the Bachelor in Psychology program participated voluntarily for educational purposes, receiving partial course credit. The current dataset was also used as one of three studies, with a very different research question (Sample 3; Franzen, Sadikaj, Moskowitz, Ostafin, & aan het Rot, 2018). We excluded 21 participants who missed more than 3 EMA days or dropped out voluntarily, and six participants who still lived in their parental home. The remaining 82 participants (see Table 1) were aged 18–25 years. The median time to travel to their parental home was 2.5 hours (range 0.1–13). The median time since moving out was 5 months (range 0.5–84). Due to data skewness, log‐transformed values were used for the latter two variables.

**Table 1:** Relevant participant data

	Dutch students	International students
N	34	48
Gender ratio (% female)	69	65
Age in years, *M* (*SD*)	20 (1.8)	20 (1.2)
Living situation (%)		
With friends or roommates	88	87
With a romantic partner	6	4
Alone	6	9
Months since moving out, median (range)	5 (1–60)	6 (0.5–84)
Travel hours to parental home, median (range)***	1.2 (0.1–3.0)	3.6 (0.4–13)
UHS‐8 score, *M* (*SD*)*	1.5 (0.8)	1.8 (0.8)
Number of completed EMA questionnaires, *M* (*SD*)	46 (22)	45 (16)
Averaged momentary homesickness, *M* (*SD*)**	0.20 (0.3)	0.55 (0.6)

*Note*: Most international students were from Germany. UHS‐8 = Utrecht Homesickness Scale, 8‐item version; EMA = ecological momentary assessment.

*p < .05. **p < .01. ***p < .0001.

### Measures

*Utrecht Homesickness Scale* (van Vliet, 2001). This self‐report questionnaire was used to assess homesickness in the past 4 weeks. There are 5 subscales, based on 20 items rated from 0 (“not”) to 4 (“very strong”). Following Stroebe et al. (2016), we limited homesickness to the feeling of missing home, excluding possible correlates such as adjustment difficulties, loneliness and rumination. The eight items of the Missing family and Missing friends subscales were averaged as an indication of proneness to homesickness. The Cronbach coefficient α for the Utrecht Homesickness Scale (UHS)‐8 was .85, indicating high internal consistency.

*Ecological momentary assessment*. We used the event‐contingent recording method developed by Moskowitz and Sadikaj (2012) to sample social interactions from daily life. A social interaction was defined as a conversation lasting a substantial amount of time, usually at least 5 minutes. Participants were instructed to complete standardised questionnaires right after each interaction, using the online software TEMPEST (Batalas & Markopoulos, 2012). TEMPEST provides participants with a web application that emphasises usability on mobile devices, eliminating the necessity to mail in questionnaires, and providing better compliance monitoring. Participants could also complete questionnaires when offline; these data were sent to the server whenever participants reconnected.

Each questionnaire included a list of affect items (Diener & Emmons, 1984) with “homesickness” added for the purpose of the present research. Participants used a scale from 0 (“not at all”) to 6 (“extremely”) to indicate how they felt during an interaction. Ratings on the items worried/anxious, frustrated, angry/hostile, unhappy and depressed/blue were averaged to create an unpleasant affect score. Ratings on the items happy, pleased, joyful and enjoyment/fun were averaged to create a pleasant affect score. The questionnaire contained additional items not considered for the present study.

### Procedures

Students interested in the study scheduled a meeting with a research assistant via an online system. Upon arrival in the university laboratory, they read a study information sheet and discussed it with the assistant. The study rationale was explained in terms of obtaining data on social interactions in real time rather than by retrospection; homesickness was not mentioned as a variable of interest. After providing written informed consent, participants completed several questionnaires including the UHS.

Participants received detailed EMA instructions and were asked to complete as many questionnaires as possible for 14 days. Some participants voluntarily continued data recording until the second meeting, which took place within a week after the EMA period and involved completing several more questionnaires (not considered here). Participants completed a mean number of 15 EMA days (*SD* = 2.4) and 45 questionnaires (*SD* = 19).

### Data analyses

We removed questionnaires completed within 3 hours of drinking alcohol (9%). The remaining EMA data included 3365 events. For H1, momentary homesickness levels were averaged across interactions for each person separately and compared across groups using a *t*‐test. For H2 and H3, we used mixed models with maximum likelihood estimation in SAS 9.3 (SAS Institute: Cary, NC, USA). The degrees of freedom for *F* tests were determined according to the method by Kenward and Roger (1997). All models included a random intercept and the default error covariance matrix. The significance level was .05 (see the Results section for more details).

## RESULTS

Standardised UHS‐8 scores positively predicted momentary homesickness, *β* = .27, *F*(1,83.1) = 26.59, *p* < .0001. This effect remained after adding nationality (Dutch vs. international) as a moderator, with no significant UHS‐8 by nationality interaction, *F*(1,83.1) = 2.79, *p* = .09. Thus, students with higher proneness homesickness reported more momentary homesickness.

### Hypothesis testing

**H1: international students experience more homesickness than Dutch students**

UHS‐8 scores and averaged momentary homesickness levels were both higher among international students (see Table 1). Thus, international students experienced more homesickness.

**H2: homesickness is higher when away from parental home and when interacting with people known before moving away**

Table 2 summarises the outcomes of the multilevel analyses. Models with single predictors included all events and revealed effects for all four included contextual variables thought to reveal during which interactions participants would be physically away from home but interacting with individuals known before moving out. The effect for Location (signifying where the interaction took place) suggested that levels of homesickness were highest when interactions took place at the current residence and lowest when interactions took place at the parental home. The effect for Contact Type (signifying whether participants knew their interaction partner from before moving away) indicated the highest homesickness levels during interactions with previously‐known people and the lowest levels during interactions with previously‐unknown people. The effect for Relation (clarifying partner roles) suggested that participants felt most homesick during interactions with parents and least during interactions with work supervisors and university teachers. The effect for Contact Mode (clarifying how the interaction took place) indicated that levels of homesickness were highest during video‐chats and lowest when interacting in person.

**Table 2:** Multilevel models for examining contextual factors influencing homesickness

Predictors	Proportion of events (%)	Single predictor in model	All predictors in model^a^
Location		*F*(4,3328) = 14.96***	*F*(4,2300) = 9.20***
Current home	40	0.53 (0.06)***	0.95 (0.08)***
Parental home	12	0.20 (0.07)***	0.54 (0.10)***
School/work	21	0.34 (0.06)	0.81 (0.08)
Recreation	9	0.28 (0.07)	0.77 (0.10)
Other	18	0.38 (0.07)	0.82 (0.09)
Contact type for primary other		*F*(3,3344) = 3.55*	*F*(2,2335) = 1.80
Known, from city of residence, Groningen	14	0.38 (0.06)	0.80 (0.09)
Known, not from city of residence, Groningen	19	0.50 (0.07)**	0.71 (0.08)
Unknown, from city of residence, Groningen	36	0.36 (0.06)**	0.82 (0.08)
Not applicable (group interaction)	31	0.41 (0.06)	—
Role of primary other		*F*(9,3322) = 3.84***	*F*(8,2309) = 5.01***
Supervisor/teacher	3	0.20 (0.10)***	0.62 (0.11)
Coworker/fellow student	7	0.45 (0.08)	0.81 (0.09)
Supervisee	<1	0.61 (0.26)	1.04 (0.26)
Acquaintance	2	0.45 (0.12)	0.82 (0.12)
Friend	28	0.37 (0.06)	0.66 (0.08)
Romantic partner	11	0.33 (0.07)	0.55 (0.08)***
Parent	9	0.62 (0.07)***	0.96 (0.09)***
Sibling	2	0.45 (0.11)	0.85 (0.12)
Other	7	0.37 (0.08)	0.69 (0.09)
Not applicable (group interaction)	31	0.41 (0.06)	—
Contact mode		*F*(2,3320) = 98.89***	*F*(2,2299) = 57.87***
In person	91	0.34 (0.07)***	0.31 (0.07)***
Via phone	6	0.84 (0.08)	0.73 (0.09)
By video chat	3	1.29 (0.10)***	1.29 (0.12)***

*Note*: Point estimates expressed as *M* (*SE*). For each predictor, the asterisks besides two *M* (*SE*) values indicate where the largest difference was found and the significance of this difference.

a Model included 2338 of 3365 events (69%).

*p < .05. **p < .01. ***p < .001.

As these findings may have been confounded by some combinations being more common than others (e.g. interactions with parents were least likely to take place in person), a model including all four contextual variables was also run. Group interactions were by necessity excluded, leaving 2338 events in the model. Nonetheless, the main effects for Location, Role and Contact Mode remained significant: participants felt most homesick in their current residence, regardless of whom they were interacting with and how; during interactions with parents, regardless of where they were and what the mode of interaction was; and when chatting by video, regardless of where and with whom they chatted. Homesickness was higher away from home and during interactions with parents, which supports our hypothesis.

**H3: feeling homesick is associated with more unpleasant and less pleasant affect**

The outcomes of the following multilevel analyses are based on models that included momentary homesickness (within‐person centred), overall mean momentary homesickness (grand‐mean centred) and their interaction as predictors. The contextual factors examined when testing H2 were included as covariates.

*Pleasant affect*. There was a negative effect for momentary homesickness, *β* = −.09, *F*(1,3283) = 8.51, *p* = .004. The momentary homesickness by overall mean momentary homesickness interaction was not significant, *F*(1,3283) = 1.19, *p* > .27.

*Unpleasant affect*. There was a positive effect for momentary homesickness, *b* = .22, *F*(1,3286) = 117.06, *p* < .0001. In addition, the momentary homesickness by overall mean momentary homesickness was significant, *F*(1,3284) = 5.68, *p* = .02. The slope of the effect of momentary homesickness on unpleasant affect was significant for participants with lower overall mean momentary homesickness, *b =* 0.28, *t*(3285) = 7.00, *p* < .0001 and for participants with higher overall mean momentary homesickness, *b* = 0.17, *t*(3286) = 9.16*, p* < .0001. The difference between the slopes was significant, indicating that when participants with lower overall mean momentary homesickness were homesick, they experienced more unpleasant affect than when participants with higher overall mean momentary homesickness were homesick.

## DISCUSSION

We examined homesickness in 1st‐year university students. In line with H1, international students reported more proneness to homesickness and more momentary homesickness than Dutch students. Both homesickness measures were moderately correlated, indicating that students who rated themselves as being more homesick over the past 4 weeks continued to experience more homesickness momentarily. This corresponds with earlier findings among children at camp (Thurber & Sigman, 1998).

When testing H2, we found that students felt most homesick during interactions at their current residence, with parents and via video‐chat. This is consistent with the idea that homesickness may be triggered especially in contact with non‐present attachment figures, and/or when university‐related distractions are less evident.

Finally, when testing H3, we found that students indeed experienced less pleasant and more unpleasant affect when feeling homesick. This might reflect a potential mechanism in the development of mental health problems among homesick students. In addition, students with lower overall homesickness experienced more unpleasant affect the moment they did feel homesick. So, in individuals who generally experience little homesickness, homesickness may elicit other negative feelings and “hit harder,” or homesickness may mostly come up in those moments when feeling sad, anxious or angry in the new environment.

### Limitations

Overall levels of homesickness may have been low in the current sample, as people who are more easily homesick may be less likely to leave home (van Vliet, 2001). Further, mental health problems were not assessed, limiting our conclusions to momentary affect. Furthermore, no causal relations can be drawn from our data. For instance, the finding that students experience more homesickness when interacting with parents may be due either to students contacting their parents when feeling homesick or to a (video or phone) interaction with “home” eliciting homesickness.

### Opportunities for future studies

By examining homesickness in daily life using EMA, we have highlighted the role of social context. Future research could examine situational factors in more detail. From attachment theory one might predict that homesick insecurely attached individuals would react with unpleasant affect when interacting with attachment figures (thereby worsening their distress), while homesick securely attached individuals may experience pleasant affect during such interactions (thus enabling them to cope better with distress). Attachment measures could be included in future studies.

Future studies could also examine the antecedents and consequences of momentary homesickness, to find out when homesickness might elicit social interaction or result from it. Also, signal‐contingent recording of affective states could be employed. Compared to event‐contingent recording data, signal‐contingent recording data are better suited to time‐series analysis, which allows for examining both concurrent and lagged associations between homesickness and affect. Signal‐contingent recording would also allow for assessing homesickness in non‐social contexts (i.e. when people are alone, when they obviously feel homesick at times too).

In conclusion, our study provides preliminary data supporting the merits of studying momentary homesickness. Identifying high‐risk homesickness situations may aid the development of (preventive) interventions for people at risk of homesickness‐related health problems.
